# Mercury Bonding to Xerogel: The Interface Fractal Dynamics of the Interaction between Two Complex Systems

**DOI:** 10.3390/gels9080670

**Published:** 2023-08-18

**Authors:** Maria-Alexandra Paun, Vladimir-Alexandru Paun, Viorel-Puiu Paun

**Affiliations:** 1 Division Radio Monitoring and Equipment, Section Market Access and Conformity, Federal Office of Communications (OFCOM), 2501 Bienne, Switzerland; 2 Five Rescue Research Laboratory, 75004 Paris, France; vladimir.alexandru.paun@ieee.org; 3 Physics Department, Faculty of Applied Sciences, University Politehnica of Bucharest, 060042 Bucharest, Romania; viorel.paun@physics.pub.ro; 4 Academy of Romanian Scientists, 50085 Bucharest, Romania

**Keywords:** complex systems, fractal-type objects, bi-stable behaviors, SEM images, fractal parameters

## Abstract

This study describes novel solid substances founded on chitosan and TEGylated phenothiazine that have a high ability for hydrargyrum recovery from watery liquid solutions. These compounds were taken into account, consisting of two distinct entity interactions inside of the classic fractal dynamics conjecture of an “interface”. They were assimilated through fractal-type mathematical objects and judged as such. The bi-stable behavior of two fractally connected objects was demonstrated both numerically and graphically. The fractal character was demonstrated by the fractal analysis made using SEM images of the xerogel compounds with interstitial fixed hydrargyrum. For the first time, SEM helped to verify such samples from two distinct bodies, with the multifractal parameter values being listed in continuation. The fractal dimension of the rectangular mask is D_1_ = 1.604 ± 0.2798, the fractal dimension of the square mask is D_2_ = 1.596 ± 0.0460, and the lacunarity is 0.0402.

## 1. Introduction

The so-called heavy metals, which have intermediate properties between those of metals and nonmetals, are known as metal chemical elements themselves and have a comparatively large density in comparison to water density, which is what the appellative heavy refers to [1]. In the well-established hypothesis that toxicity and heaviness are capitally connected, the hard/heavy metals also cover the generic category of metalloids, such as silicon, polonium, and arsenic, that are capable of high toxicity and need to be reduced in terms of human exposure level [2]. In the same category of disadvantages, the so-called pure metals can also be introduced, as they are directly responsible for the damage they produce. In this article, we will only deal with one of them, namely mercury, which is fixed interstitially in compounds with xerogels. However, we remind you that the metals mentioned above do not have well-established biological functions and are, thus, taken into account as non-essential active metals from this point of view [3,4]. The important heavy metals exercise some biochemical, biophysical, and physiological major functions in vegetable and animal organisms.

Thus, these substantial constituents of integrated basic enzymes have or manifest significant roles in various complex reactions, the most important being oxidation–reduction. Among the original metals, we mention Copper, which participates as a necessary cofactor in some oxidative stress-connected enzymes, such as ferroxidases, monoamine oxidase, catalase, and specific dopamine β-monooxygenase [5]. Thanks to the exponential growth in ecological awareness and the global public health correlated with the territory surrounding contamination concerning the mentioned metals, these problems have become among the most important contemporary issues. In addition, human subjection to the alleged contamination has drastically increased as a result of an exponential magnification of their use in diverse sectors, such as agriculture, industrial, technology, and domestic [6,7,8,9].

The main problem is the toxicity of these chemical elements and the respective metals in general. Thereby, the degree of toxicity presented is a function of several factors, from which we can cite a few that are the most important: the chemical species, effective dose, and the reason for exposure or contamination route, as well as genetics, gender, age, particular nutritional status, and individual exposure levels [10,11,12]. On account of their great degree of toxicity, cadmium, chromium, arsenic, mercury, and lead are considered the pre-eminent metals that are of social relevance in terms of health. The listed metallic elements are considered everywhere as being toxic and inflicting manifold damage to the body, even with a decrease in exposure levels. In addition, they have been declared principal sources of human carcinogens by the authority of the Environmental Protection Agency (EPA) and the International Agency for Research on Cancer (IARC), which are both based in the United States of America.

Mercury (Hg), popularly referred to as quicksilver, is a chemical element that is in a liquid state under normal conditions of pressure and temperature and is a Group 12 metal (the same group as zinc) according to the modern periodical table. Mercury belongs to the category of heavy metals and the transition element series, respectively [13,14,15]. It is a unique element for the fact that it can be found in three distinct forms (elemental, organic, or inorganic) in nature, each with its own toxicity profile [14]. In addition, mercury has two different forms of cationic existence; more specifically, the first is called mercurous (+1 oxidation state), and the second is called mercuric (+2 oxidation state). It has a high pressure concerning its vapors (with which it is in equilibrium) and is released in the form of vapors in the environment surrounding it. Humans are subjected to serious accidents in terms of intoxication, with most presenting forms of mercury intake, such as those produced by agricultural and industrial works, contaminated food, disease prevention actions, dental repairs, and professional activities. The important chronicle sources of lower-stage mercury exposure are dental amalgam compounds and the consumption of fish as food. The particularly absorbed mercury assortments/varieties are elemental mercury (Hg^0^) and methyl mercury compounds (MeHg). Dental amalgam compounds include upwards of 50% mercury in its elementary form [12]. As soon as the mercury has been absorbed, it remains in the body for a long time because it has a reduced elimination rate. A considerable part of the absorbed amount is collected in the neurological tissues, kidneys, and liver. All mercury varieties have a high degree of toxicity that act in terms of nephrotoxicity, gastro or intestinal toxicity, and neurotoxicity on vital human organs.

The fractal analysis method [16] is a quantitative procedure of picture investigation that is founded on three accepted numerical characteristics, such as fractal dimension, lacunarity, and respectively, succolarity. The fractal dimension is the estimate that distinguishes how much a fractal geometric object replenishes the volume at its disposal (available volume). Practically, this dimensional measure is a consecrate size that does not depend on the chosen geometric scale and by the translation process practiced or rotational process. The second characteristic, lacunarity, accounts for the quantification of the proportion of holes and their frequency of appearance in the image. The third characteristic, called succolarity, establishes what quantity of a physically distinct (representative) fluid may run over a frame, using the pixel group as the barrier, which has a well-established coloring of white or black in, e.g., 2D image assessments [17]. The latter is not relevant to our study, so we will not discuss it further.

The objective of the work is to demonstrate that the behavior of the materials formed by fixing interstitial mercury in some xerogels is multifractal. This fact is determined by a mathematical model that governs the fractal behavior of the system of two solid bodies and by the fractal analysis of the SEM images of the samples obtained. The values of the fractal parameters, the fractal dimension, and the respective lacunarity are the factors that legitimize the fractal behavior of fusion materials.

## 2. Theoretical Part

This article is the first to treat xerogel compounds with fixed mercury from the point of view of the multifractal analysis of two independent fractal mathematical objects. The most important novelties introduced in the current work are the following: the mathematical equations are new and have been successfully used to theoretically define and support a xerogel and the associated mercury as two distinct fractal objects. The fractal character was proven by the fractal analysis made on the SEM images of these compounds. The bi-stable behavior of two fractally connected objects was demonstrated both numerically and graphically.

### 2.1. Fractal Parameters

In fractal conjecture, fractal parameters, such as the fractal dimension, lacunarity, and succolarity, are currently used to enable access to the structural details of a pore material complex system in action. It is worth remembering the fact that the practical application and correct estimation of the three aforementioned characteristics are difficult due to the complicated definitions and arduous procedures of calculation [18]. In the related section, we particularly initiate the presentation of authoritative definitions and the substantial significance of the fractal characteristics and a calculus procedure founded on the box-counting process (from the images mainly, but also from other methods).

#### 2.1.1. Fractal Dimension

Heuristic fractal media may be adapted as measurable metric groups that have non-integer dimension values [16]. Thus, we can say that the non-integer Hausdorff dimension is a basic attribute of the fractal object. On the authority of Benoit Mandelbrot, the denomination of a fractal is associated with a group of objects for which the Hausdorff-Besicovitch-type dimensions are totally different from the classical topologic dimensions, with its integer values being specific to Euclidean geometry.

Let $N_{\epsilon \;}\left(A\right)$ be a minimal number of closed balls (spherical shapes), which cover well all space *A*, with ϵ being a very small positive number. We can consider that, mathematically, the object *A*(ϵ) a is a topological compact subset and has the fractal dimension *D*(*A*), or more simply denoted by *D*, if we have

(1) $$D=\underset{\epsilon \to 0}{\lim}\frac{\log\left(N_{\epsilon \;}\left(A\right)\right)}{\log\left(\epsilon^{-1}\right)}=\underset{\epsilon \to 0}{\lim}\frac{\log\left(N_{\epsilon \;}\left(\epsilon \right)\right)}{\log\left(\frac{1}{\epsilon }\right)}$$

on the condition that the limit in question exists and is finite [17].

Whereas for the zero limit, which cannot be assigned to some natural object dimensions, the mathematical dimension was equated by the algebraic fractal dimension *D* = *d* as the solution, with *d* as the slope from the graphical representation of log *N*(*ε*) as a function of log (1/*ε*). The linear curves of these graphical representations were subjected to the regression technique by the least-squares procedure, whereas the gradients of the respective curves were calculated by utilizing a conjugate gradient procedure, which is a fairly well-known iterative procedure in mathematical problem resolution [18].

#### 2.1.2. Lacunarity

A lacunarity intuitional definition refers to the fact that it is a good measure of the distribution of crevasses (gaps). More precisely, it is a reflection of the volume of gaps/holes (the portions without physical material present) when compared to the entire available volume [19]. For a fractal object, the more it is full of defects/holes (i.e., lacunar), the more numerous its gaps and, furthermore, these are apt to be bigger, meaning that this includes special wide geometric figures (disks or spheres). In the fractality assumption, the lacunarity concept relates and defines/explains (mathematically) the presence of current holes (that is why it is surnamed after a porous texture), and on top of that, we find it to be synonymous with the quantitative radiography of the “correct or real texture” [20]. We see that the poor inhomogeneity stage, as well as the (2D) translational and (3D) rotational invariance of the frame plain surface, confirm the fact that the rotations performed modify the initial context in an insignificant manner [21,22,23]. In conclusion, insufficient lacunarity proves the superior homogeneity of the evaluated pictures [24,25,26,27].

### 2.2. Fractal Mathematical Model

In multiplex network dynamics, nonlinear behavior and disorderliness act as systemic and functional phenomena of irregular motions and critical instabilities. The interactions between the constituent fractal objects of complex aggregates offer reciprocal limitations and local-topical-global connection comportment [28,29].

Let the following differential equation be accepted as general law:

(2) $$\frac{dQ_{t}}{dt}=(Q_{i}-Q_{t})+\frac{AQ_{t}}{1+Q_{t}{}^{2}}$$

in the “interface” dynamics depiction case of the reciprocation of two complex independent systems. The two bodies in the complex system can be undoubtedly approved as fractal-type objects via the use of mathematics. In Equation (2), *Q_t_* is the transmitted fractal field variable, *t* is the temporal variable, *Q_i_* is the incident fractal field variable, and *A* is a parameter independent of the fractality degree in the resolution scales space, by which it is possible to alternate the distinct selfstructuring regimes of individually existent complex systems. This particular result was achieved from the general differential equation in the scale resolutions space:

(3) $$\frac{dQ_{t}}{dt}\approx \bar{A}+\bar{A_{t}}Q_{t}=\frac{A_{1}Q_{t}{}^{3}+A_{2}Q_{t}{}^{2}+A_{3}Q_{t}+A_{4}}{\bar{A_{3}}Q_{t}{}^{2}+\bar{A_{2}}Q_{t}+\bar{A_{1}}}$$

by operating with the identities

(4) $$A_{1}=-1,\;A_{2}=Q_{i},\;A_{3}=-\left(1+A\right),\;A_{4}=Q_{i},\;\bar{A_{1}}=1,\;\bar{A_{2}}=0,\;\bar{A_{3}}=1$$

and $\bar{A_{t}}$ is a proportionality constant. In addition, we can mention that the variables *Q_i_*, *Q_t_*, and *A* play the role of dimensionless variables. Regarding Equation (2), it is specified that, at all scale resolutions, the first derivative with respect to the time of the transmitted fractal field variable, (d*Q_t_*)/d*t*, is determined both by the difference between the incident fractal field variable and the transmitted fractal field variable, (*Q_i_* − *Q_t_*), and by another important term: the saturation component, noted as (*AQ_t_*)/(1 + (*Q_t_*)^2^).

In these given circumstances, it can be considered that fractal dynamics systems are correctly represented by the scale resolutions space by the equality between the first-order derivatives below:

(5) $$\frac{dQ_{t}}{dt}=-\frac{dV\left(Q_{t}\right)}{dQ_{t}}$$

The above equation can be arranged and integrated as follows:

(6) $$dV\left(Q_{t}\right)=-\left(\frac{dQ_{t}}{dt}\right)dQ_{t}\;\int^{\;}dV\left(Q_{t}\right)=-\int^{\;}\left(\frac{dQ_{t}}{dt}\right)dQ_{t}$$

which gives the result below, using the appropriate integration limits.

(7) $$V\left(Q_{t}\right)=-\int_{0}^{Q_{t}}\left(Q_{i}-Q_{t}{}^{\prime }-\frac{AQ_{t}{}^{\prime }}{1+Q_{t}{{}^{\prime }}^{2}}\right)dQ_{t}{}^{\prime }=-Q_{i}Q_{t}+\frac{Q_{t}{}^{2}}{2}+\frac{A}{2}\ln\left(1+Q_{t}{}^{2}\right)$$

*V*(*Q_t_*) is noted as a fractal potential function, which is a function that features as a significant category in dynamic fractal systems, more recently generically referred to as “gradient fractal systems” [17,21].

**Figure 1:**
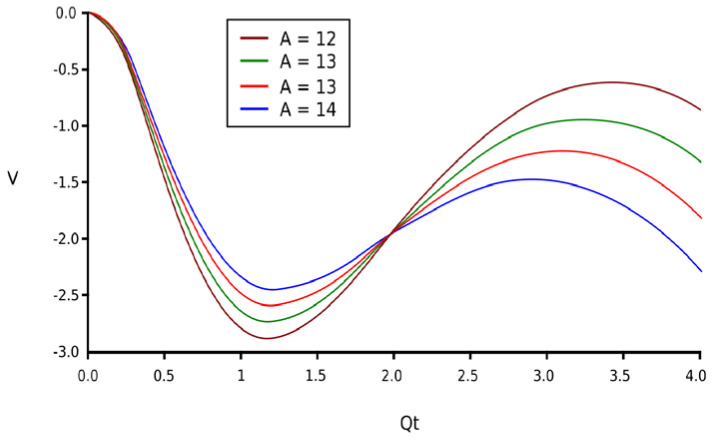
The family of curves of the fractal potential for several proportionality constants.

Equation (7) is graphically represented in Figure 1, having four parameters with different values. Now, it can be mentioned that the circumstance that *V*(*Q_t_*) describes is comportment covered by a pair of potential wells. The comportment in discussion admits that, from the perspective of progress to equilibrium and, obviously, the steadiness of the equilibrium circumstances, the fractal system governed by Equation (7) is of a similar manner to that of the fractal oscillator related to the fractal potential formula. The certainty values of *V*(*Q_t_*) can be considered actual states/positions of equilibrium, but at the same time, the maximum limits are within the domain of the values, which can be assimilated with unsteady equilibrium positions.

Regarding the fractal potential function obtained above, we may say, mathematically, that it depends on three dimensionless variables, *Q_i_*, *Q_t_*, and *A*, when also considering the possible values of the constant *A* in the range of interest:

(8) $$V\left(Q_{t},\;Q_{i},\;A\right)=\frac{Q_{t}{}^{2}}{2}-Q_{i}Q_{t}+\frac{A}{2}\ln\left(1+Q_{t}{}^{2}\right)$$

Figure 1 shows the fractal potential function of four curves, *V* = f(*Q_t_*), for four values of the constant *A*.

**Figure 2:**
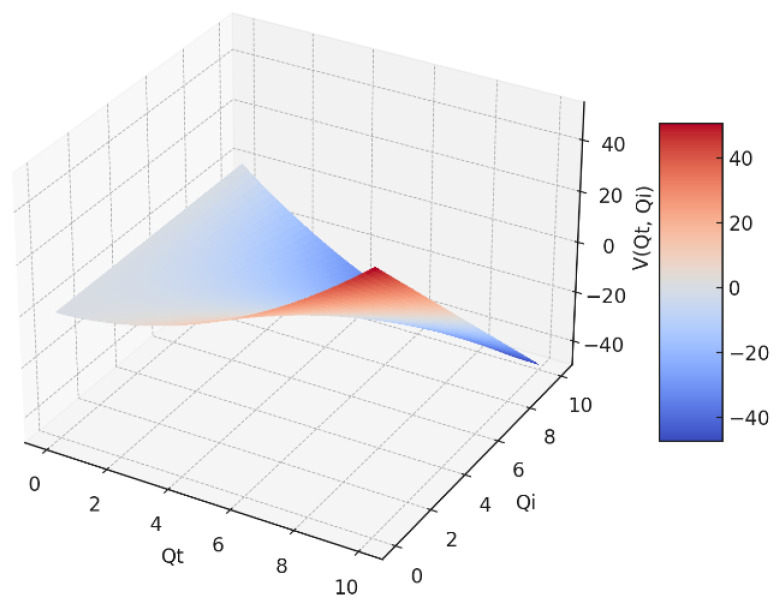
The fractal potential in a 3D representation as a function of *Q_i_* and *Q_t_*.

In Figure 2, we have a 3D representation of the fractal potential *V* = *V*(*Q_t_*,*Q_i_*) as a function of two variables: the transmitted fractal field variable (*Q_t_*), and the incident fractal field variable (*Q_i_*), respectively.

**Figure 3:**
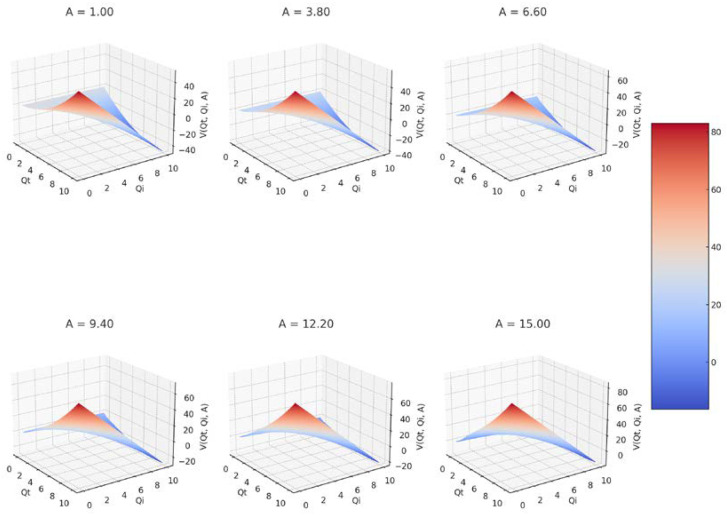
A 3D representation of fractal potential for six values of constant *A*.

Six 3D graphs of the fractal potential as a function of *Q_i_* and *Q_t_*, each for a different value of the constant *A* are presented in Figure 3.

Figure 3 presents six 3D graphs for six values of constant *A*: *A* = 1.00, *A* = 3.80, *A* = 6.60, *A* = 9.40, *A* = 12.20, and *A* = 15. The colors located on the color bar on the right signify the value of the fractal potential included in the range from −20 to 40 for the first two, respectively, from −20 to 60 for the next three, and from −20 to 80 for the last graph.

**Figure 4:**
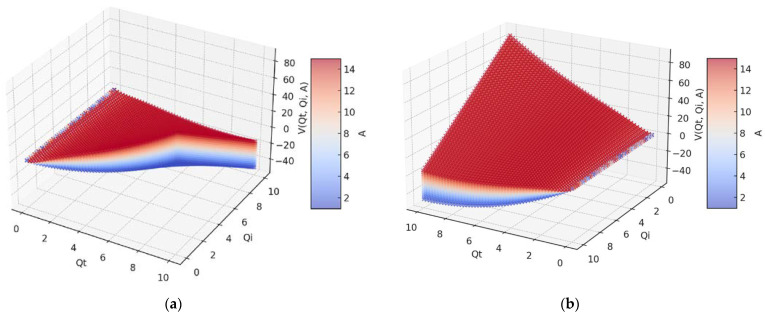
A 3D representation of fractal potential for 14 values of constant A. (**a**) central position, (**b**) rotation by 30 degrees.

In Figure 4, we have two 3D graphs of the fractal potential, (a) and (b), of three variables *V* = *V*(*Q_t_*,*Q_i_*,*A*), with two distinct orientations concerning the axes of the variables *Q_t_* and *Q_i_*. Constant *A* has positive values between 1 and 14, which can be seen on the colored bar on the right with distinct colors from blue to red-brown.

What is remarkable in these 3D representations of the curves considered by the four variables is the fact that the fourth variable gives volume to the representation itself, being evaluated according to the color marked on it, from blue to red-brown.

The stationary fractal behavior of the complex system of two bodies can be analyzed at all resolution scales in the cases generated by the equation d*Q*/d*t* = 0. Practically, this happens when discussing the implications of the solutions of the previously mentioned equation, which establishes the local extrema of the real function *Q* = *Q*(*t*). We mention that this is the only way we can highlight the perspectives of an evolution over time of the system towards equilibrium, respectively, and the correct establishment of the corresponding individual states and their stability. Next, we look for the stationary behavior of the fractal complex system, as described by Equation (2): $\frac{dQ_{t}}{dt}=0,$ which involves studying the function *Q_i_* = F(*Q_t_*) at all scale resolutions:

(9) $$Q_{i}=Q_{t}\left[1+\frac{A}{1+Q_{t}{}^{2}}\right]$$

**Figure 5:**
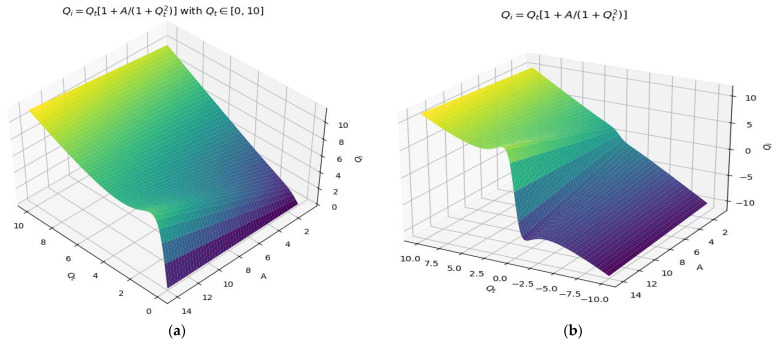
A 3D representation of the incident fractal field *Q_i_*, and the function of two variables, *Q_t_* and *A*, for two variation domains of *Q_t_*. (**a**) transmitted fractal field between 0 and 10, (**b**) transmitted fractal field between −10 and 10.

In Figure 5a,b, 3D representations of the incident fractal field *Q_i_*, the function of two variables, *Q_t_*, the transmitted fractal field, and the constant *A*, respectively, for the two variation domains of *Q_t_* can be seen, with one explicitly noted over the other (see graph a).

In Figure 5a,b, a 3D representation of the incident fractal field *Q_i_*, the function of two variables, *Q_t_*, the transmitted fractal field, respectively, and the constant *A* for two distinct variation domains of *Q_t_* are shown. In Figure 5a, the transmitted fractal field is $Q_{t\;}\in \left[0,10\right]$, and in (b), it is $Q_{t\;}\in \left[-10,\;10\right]$.

**Figure 6:**
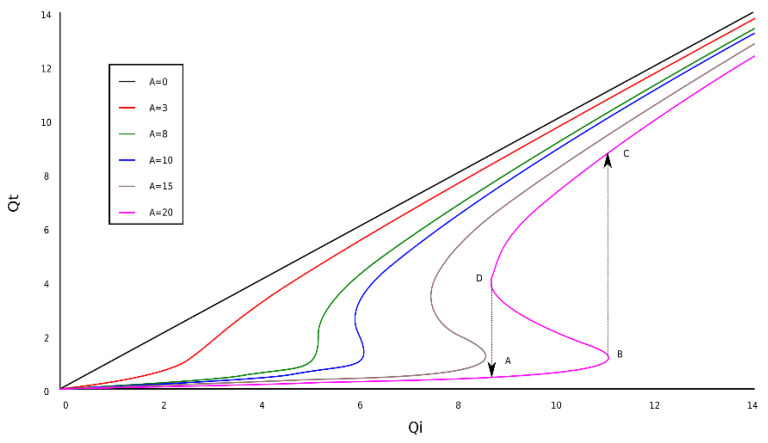
The dependence of the transmitted fractal field as a function of the incident fractal field.

Note that we have the 3D representation against the three axes denoted by (*Q_i_*,*Q_t_*,*A*). In this axis system, the surface *Q_i_* = *Q_i_*(*Q_t_*,*A*) was plotted. This is a double wrinkle catastrophe-type multifractal field area in terms of 3D dimensions (Figure 5) (see the mathematical standard event described in [25,27]). It can be remarked that the reversal curves shown in Figure 6 are correctly assimilated, with some transitions effectuated into the multiscale ideal space [29,30].

In Figure 6, the graphical representation of transmitted fractal field dependence as a function of the incident fractal field, *Q_i_*, into a situation on fractal bi-stability comportment is shown.

The curves $Q_{t}=F\left(Q_{i}\right)$ for six values of *A* (Figure 6) show a maximum and a minimum when A attains certain values. The points marked with *A*, *B*, *C*, and *D* indicate the presence of bi-stability. Practically, this is represented by the *Q_t_* = f(*A*, *Q_i_*) variation.

These can be found by canceling the derivative of Equation (9), respectively, $\frac{dQ_{i}}{dQ_{t}}=0.$ We, thus, have the equation

(10) $$1+\frac{A\left(1-2Q_{t}{}^{2}\right)}{{\left(1+Q_{t}{}^{2}\right)}^{2}}=0\;\;\;\;\mathrm{or}\;\;\;\;\frac{Q_{t}{}^{4}+\left(2-A\right)Q_{t}{}^{2}+A-2}{{\left(1+Q_{t}{}^{2}\right)}^{2}}=0.$$

The above equation is null if and only if

(11) $$Q_{t}{}^{4}+\left(2-A\right)Q_{t}{}^{2}+A-2=0$$

Equation (11) is a bi-quadratic equation, which has four roots, these being the following:

(12) $$Q_{t}{}_{1,2}=\pm \frac{\sqrt{-\sqrt{A\left(A-8\right)}+A-2}}{\sqrt{2}}$$

and

(13) $$Q_{t}{}_{3,4}=\pm \frac{\sqrt{\sqrt{A\left(A-8\right)}+A-2}}{\sqrt{2}}.$$

**Figure 7:**
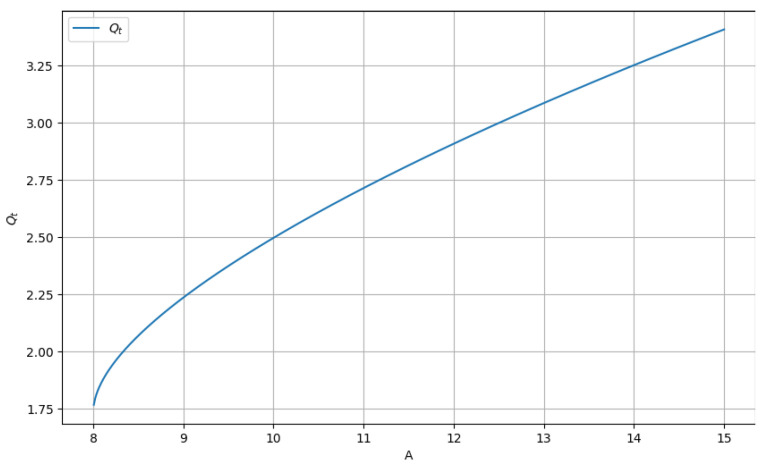
The graph of *Q_t_* as a function of constant A.

In Figure 7, we have the graphical representation of the real part and the positive variant of Equation (11).

The fractal potential as a function of the constant *A*, *Q_t_* = f(*A*) for values of *A* greater than 8, can be found in Figure 7. The value of the potential *Qt* increases rapidly with the increase in the constant *A*.

Note: It is observed that at least one studied equation root can be a complex number (that is, not a real number). The necessary condition to have a real solution is *A* being greater than or equal to 8 (*A* ≥8).

## 3. Results and Discussion

In this paper, the deep fractal nature of the association between a xerogel and interstitial fixed mercury has been demonstrated by using two methods. The first refers to the writing of mathematical formulas according to a formalism of two distinct fractal objects acting together as a unitary whole and as a single fractal body. The second method is to highlight the fractal character of the resulting SEM body images by introducing and fixing mercury atoms in the xerogel structure as the basis for processed chemical mercury deposition. The possible application of the present research is the utilization of the bi-stable behavior of two fractally connected objects. Bi-stable behavior is associated with the smectic and cholesteric phases, both of which have, in completely different ways, translational symmetries added to nematic-like orientational order.

For an exact comparison to a familiar xerogel, more chitosan xerogel reference specimens were prepared under similar conditions to the hydrogels mentioned in paragraph 5, which were labeled with the *X* index. More exactly, we have some SEM images of the specimens that were experimentally realized, images of several different xerogels, and images of one xerogel with recovered mercury.

### Fractal Analysis of SEM Image

The SEM image scale bar (located on the lower right side) in Figure 1a has a length of 100 microns, and Figure 1b has a length of 20 microns. The HV (high voltage power supply) was 5 kv.

**Figure 8:**
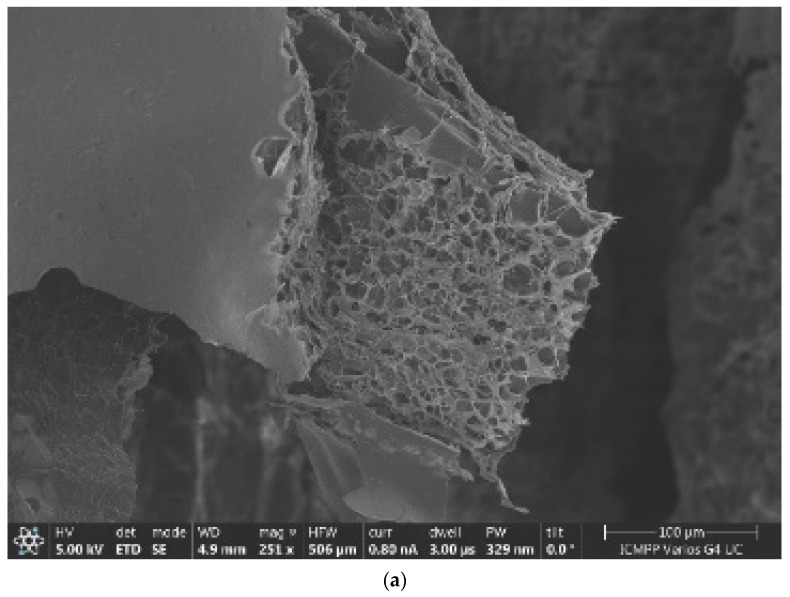

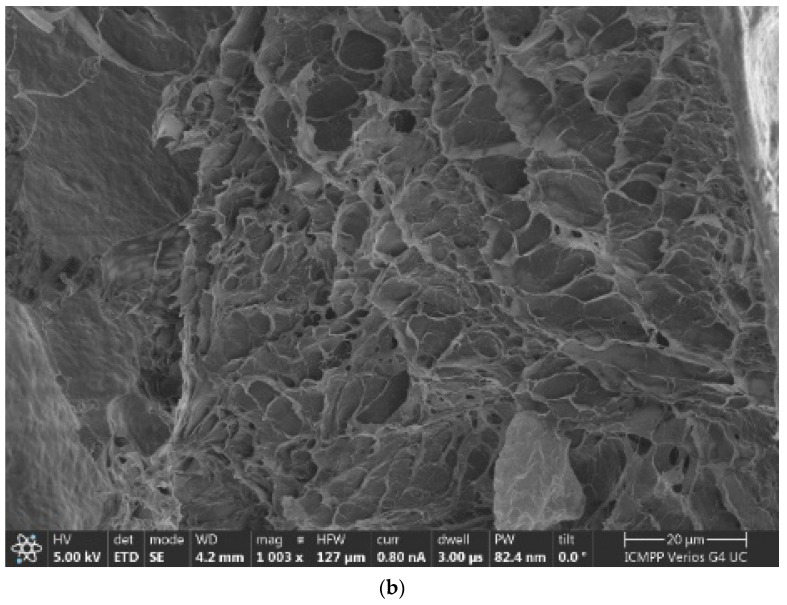
(**a**) Original image of 6X sample; (**b**) 6X-Hg sample image.

In Figure 8a, we have the 6X SEM image of a xerogel; in Figure 8b, we have the 6X-Hg SEM image of the same xerogel with interstitial mercury obtained from the mercury recuperation experiment [7].

Note that the SEM magnification (M) is defined as the ratio of the length measured from the SEM monitor—Lm—to the same length measured on the sample—Ls. M equals Lm over Ls. The length measured can be anything from the size of a single pixel all the way up to the entire horizontal or vertical field of view. A detector for secondary electrons, which is standard for all basic SEMs, records the topography of the surface under observation with a resolution in the order of 1–2 nanometers and a magnification range from 10× to 5,001,000×. In our article, in the two SEM images from Figure 8, we have the following magnifications:

(a) Magnification (power of amplification) is (251×)—251 times;
(b) Magnification (amplification power) is (1003×)—1003 times.

The magnification values are written on the black band inside, below each image.

The SEM-EDX spectrum instrument is the code name of a complex device that produces a series of operations, such as scanning electron microscopy (SEM) by energy dispersive X-ray analysis (EDX) SEM. In this way, itemized high-resolution pictures of the tested specimen are provided by rastering a centered electron fascicle across the area and the detection-backscattered (or secondary) electron target [31].

**Figure 9:**
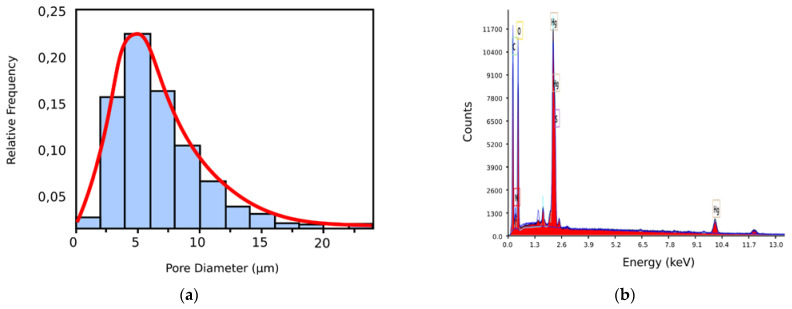
(**a**) Pore dimension histogram of 6X SEM image; (**b**) SEM-EDAX spectrum of 6X-Hg image.

In Figure 9a, we have the pore histogram for the 6X SEM image, and in Figure 9b, there is the SEM-EDX spectrum of the 6X-Hg image, confirming the presence of mercury in the investigated sample. Regarding the histogram of the xerogel in the 6X image, we notice that, at its upper limit, it looks like a normal distribution; the red line continues in the graph [29].

Owing to the fact that the classic SEM analysis revealed no obvious mercury crystallites on the material image but saw only small increases in the thickness of the pore walls (in a complicated and difficult way to be attributed to a chemical eigenstate), while the EDAX-associated instrument immediately pointed out the existence of mercury on the specimens’ surface, it was proposed that we should use the mixed apparatus. Manifestly, this allows for the fast, cheap, and nondestructive target analyses required for the characterization of surfaces (2D) and materials (3D). For the SEM-EDX spectrum of the 6X-Hg image (Figure 9b), the presence of mercury in the investigated xerogel specimen was successfully confirmed. The energy dispersive X-ray analyzer (EDX or EDA) was utilized for the identification of the individual elements in the investigated composition for the identification of mercury (Hg) and for establishing the concentration in the sample, like the one in Figure 2b. Image (b) shows the EDAX analysis of the 6X-Hg compound (the 6X hydrogel after interacting with mercury). In essence, it is a spectrum of electromagnetic emission. On the abscissa is the energy at which the atoms emit X-rays when they interact with a flow of electrons. This energy is characteristic of each atom, and thus the spectrum highlights their presence in the evaluated sample. The counts are written on the ordinate. In essence, the investigation is an elemental surface analysis, with its purpose being to highlight the types of atoms present in a sample.

**Figure 10:**
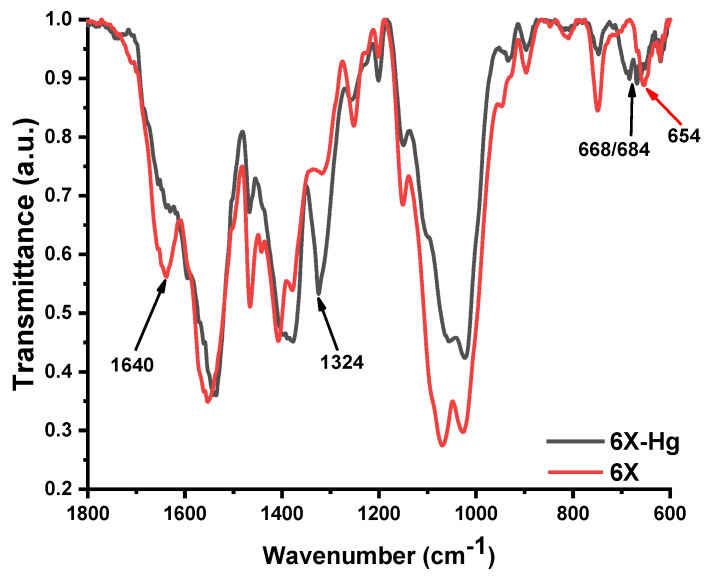
FTIR spectra of two different compounds; the first is the simple 6X xerogel, and the second is the mercury-recovery-treated xerogel, coded 6X-Hg.

In Figure 10, there are two so-called FTIR (Fourier-transform infrared spectroscopy) spectra for two distinct chemical compounds. The first compound is a simple xerogel, coded 6X, with the spectrum having a red color, and the second is a compound made of mercury bonding to xerogel, coded 6X-Hg, which has a black color.

More precisely, the FTIR spectra of xerogel 6X before (6X) and after mercury recovery (6X-Hg) are presented. In these spectra, the most evident factor was the almost total disappearance of the vibration band of the imine bond at 1640 cm^−1^. Another significant modification of the spectrum was the intensification of the stretching vibration band of the C-S-C from the phenothiazine heterocycle at 1324 cm^−1^ and the appearance of two vibration bands at 668 cm^−1^ and 684 cm^−1^, which is characteristic of the co-ordinative bonds of sulfur with mercury. In conclusion, the mercury was predominantly retained within the xerogels by co-ordination bonds with sulfur of phenothiazine, imine bonds, and the amine groups of chitosan.

**Figure 11:**
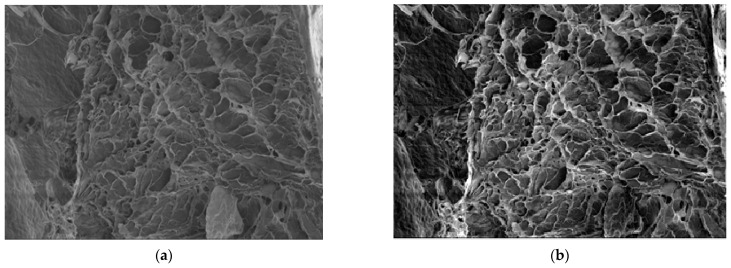
(**a**) Original image with metadata excluded; (**b**) image of interest in grayscale.

Figure 11 presents the original SEM image of the xerogel titled 6X (a) and the same computerized image to show the gray scale levels (b).

Note that according to the working procedure of SEM images, multiple images were recorded, with at least five (more than three- triplicate) per sample at two–three different magnifications. The most complete SEM image is used in this paper, which we then evaluated fractally in terms of calculating the fractal parameters, fractal dimensions, and lacunarity, respectively. We also used the image that shows the fixed presence of mercury so we could correctly consider the fractal problem of two bodies. In order to determine the fractal dimension and lacunarity, we used image processing at the pixel level, and the distances were calculated with special software that applies the 2D geometric distance formula/metric. They are average values, of course. Then, the function ln (N(r)) = f (lnr) was numerically represented, from which the slope of the fractal dimension was obtained.

**Figure 12:**
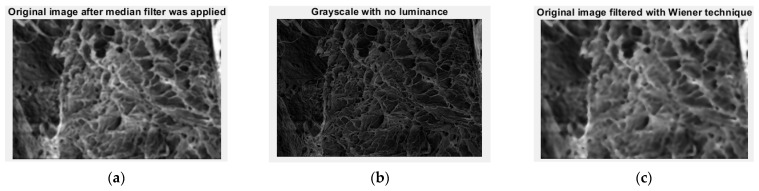
(**a**) The original image filtered with a median filter; (**b**) the image of interest in grayscale and without luminance; (**c**) original image filtered with Wiener filter.

In Figure 12, we can see the original image filtered with a median filter (a), the image in grayscale and without luminance, conjunctively (b), and the same image filtered with a Wiener filter (c).

Table 1 shows the fractal parameter values (more precisely, fractal dimension and lacunarity) calculated in cases using a rectangular mask or square mask, together with the standard deviation for both types of masks.

The fractal dimension of the rectangular mask is D_1_ = 1.604 ± 0.2798, the fractal dimension of the square mask is D_2_ = 1.596 ± 0.0460, and the lacunarity is 0.0402.

**Figure 13:**
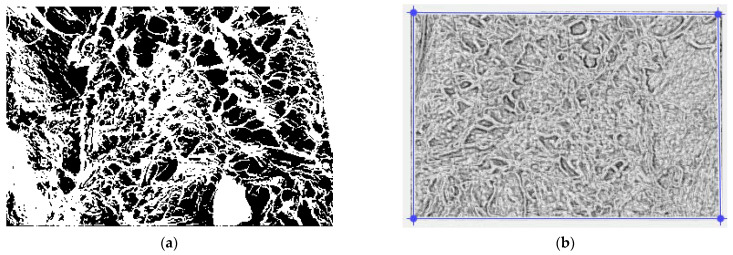
(**a**) An image of the binary version for lacunarity calculation; (**b**) defined mask for lacunarity calculation.

Figure 13a,b show the images processed in the binary version and, respectively, the definition of the applied mask used to calculate the lacunarity.

**Figure 14:**
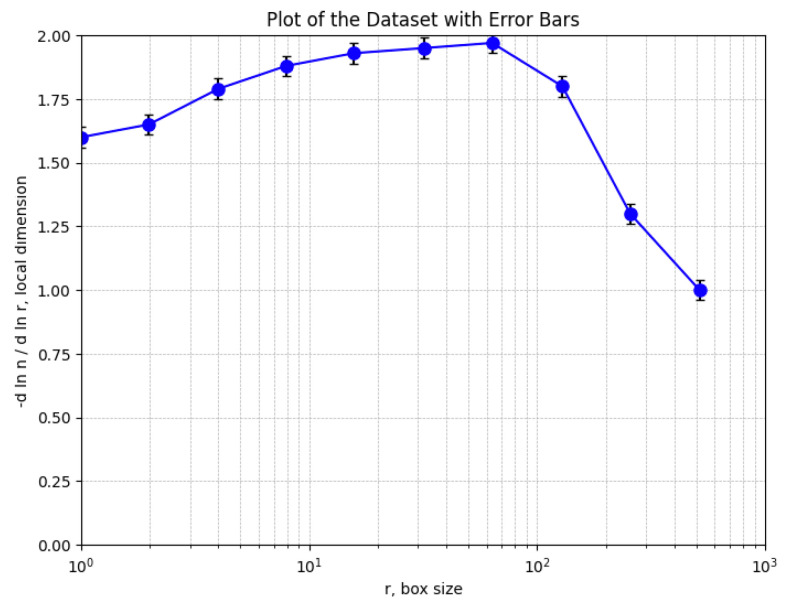
Graphical representation of local fractal dimension values, returned by box-counting.

Figure 14 shows a graphic representation of the local fractal dimension values (with error bars) as a function of box size, *r*, obtained using the box-counting method.

**Figure 15:**
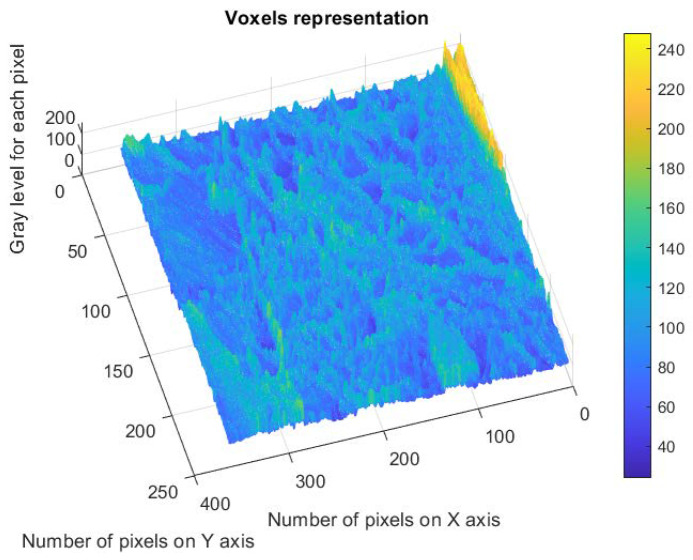
Representation of the voxels corresponding to the verified pixels in the evaluated image.

Figure 15 shows the estimated voxels of the 6X-Hg image, a 3D illustrative portrait of the voxels with the gray level values on the Oz axis, with the position of an adequate number of pixels marked on the last two plain axes and individually on the Ox axis and the Oy axis [32].

**Figure 16:**
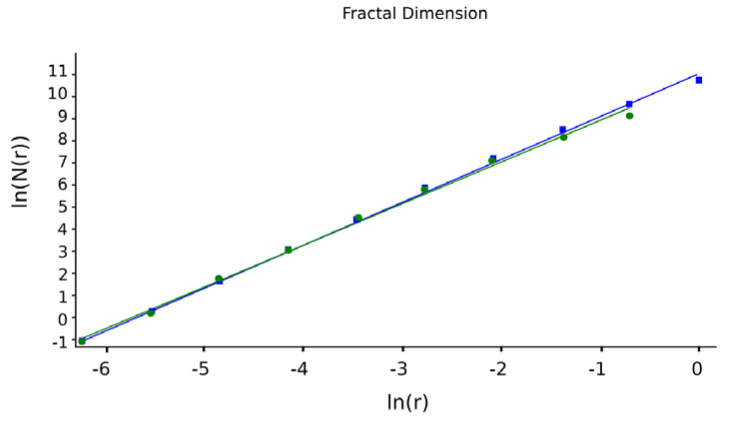
Fractal dimensions provided by multiple linear regression. Green regression line is the use of rectangular mask. Blue regression line is the use of square mask.

Figure 16 shows two lines (one green and one blue) obtained by applying multiple linear regression to some of the data equations using the function ln(*N*(*n*(*r*)) via argument ln(*r*).

The slope of these lines is equal to the fractal dimension of the studied geometric object, respective to the corresponding SEM image [18]. The values obtained for the fractal dimension are *d*_1_ = 1.71 for the green regression line and *d*_2_ = 1.74 for the blue regression line.

The results presented by us in the article have a great degree of novelty, being innovative in the development of a model of two multifractal objects treated solidly as a single complex object of a fractal nature. The innovation refers to the fact that both the fractal dimension and the lacunarity of the SEM image in the xerogel mixed compound and the interstitial fixed mercury are discussed so that the fractal character of the resulting body is demonstrated. It was proven, more precisely, that the fractal behavior in the solidarity of the compound obtained by fusing the xerogel with the fixed interstitial mercury is a local verification of the multifractal dynamics model developed by the authors.

We have not seen articles by other authors (apart from our collective) that discuss the fractal nature of SEM images of xerogels and xerogels with fixed mercury. We will make a comparison of the results with those published in the only paper we know of, which belongs to us, where a complete analysis of the morphology details of xerogels by using multifractal analysis and scanning electron microscopy images is made [30] (see the bibliography). Here, the fractal parameters of the SEM images of 5-fluorouracil released from a chitosan-based matrix are evaluated. The average values were for a fractal dimension of D = 1.8621 ± 0.0733 and a lacunarity value of Λ = 0.0385. In the current article, the average value for the fractal dimension of a rectangular mask is D_1_ = 1.604 ± 0.2798, the fractal dimension of a square mask is D_2_ = 1.596 ± 0.0460, and the lacunarity is 0.0402. The fractal dimension is much smaller, but the lacunarity is greater due to the fact that the xerogels used in the current article have more voids/interstices, such that they are able to fix mercury in larger quantities.

## 4. Conclusions

The article describes universal complex systems as being fractal-type topological objects, which, subsequently, were examined from the point of view of multiple interface dynamics as an effect of the physical interaction of these systems. It can be easily remarked that a large palette of nonlinear comportments can be consecutively assumed as having a catastrophe-type multifractal field area in three dimensions (Figure 5) and a bi-stable-type comport in Figure 6. The results presented in the article have a great degree of novelty, being innovative in the development of a model of two multifractal objects treated solidarily as a single complex object of a fractal nature. The innovation refers to the fact that both the fractal dimension and the lacunarity of the SEM image in the xerogel mixed compound and the interstitial fixed mercury are discussed such that the fractal character of the resulting body is demonstrated. It was proved, more precisely, that fractal behavior exists in the solidarity of the compound obtained by fusing the xerogel with the fixed interstitial mercury, with this being a local verification of the multifractal dynamics model developed by the authors. After the verification of the fractal character of the interaction of the two complex systems was made by analyzing the morphology of the structure fixed from the investigation of SEM images (Figure 8b), the usual fractal parameters were determined: fractal dimension and lacunarity, respectively. The calculated values are the subject of Table 1; the fractal dimension of a rectangular mask is D_1_ = 1.604 ± 0.2798, the fractal dimension of a square mask is D_2_ = 1.596 ± 0.0460, and the lacunarity is 0.0402. The fractal dimension is small, a little over 1.5, but the lacunarity is greater due to the fact that the xerogels used in the current article have more voids/interstices, such that they are able to fix mercury in larger quantities.

The chemical production of novel porous solid materials in a xerogels format, founded on phenothiazine and chitosan, which were then used as a special mercury recuperation frame, closes this study. The new substances were made possible via the hydrogelation process of chitosan, attended by the practice of lyophilization. FTIR spectra of the xerogel 6X before (6X) and after mercury recovery (6X-Hg) are presented. The mercury was predominantly retained in the xerogels by co-ordination bonds with sulfur of phenothiazine, imine bonds, and also the amine groups of chitosan.

## 5. Materials and Methods

### 5.1. The Hydrogels/Xerogels Materials, Synthesis and Characterization

The most important materials used were chitosan, with an attenuate molecular mass, tri-ethylene glycol monomethyl ether at 97% concentration, phenothiazine at 98% concentration, sodium hydride at 95% concentration, phosphorus V—oxychloride at 99% concentration, and magnesium sulfate at 99.5% concentration; all were purchased through the S.-A. Company (Sigma-Aldrich Company, St. Louis, MO, USA). The TEGylated phenothiazine means that the phenothiazine heterocycle is now replaced by TEG (Tri-ethylene Glycol); more precisely, the phenothiazine kernel is equipped with a TEG link that strengthens it. In the end, we obtained viscous chitosan, with the results verified with the help of a calibrated viscometer (Ubbelohde). A suite of three chitosan-founded hydrogels was obtained by the imination chemical reaction with a formyl derivative of tri-ethylene glycol-phenothiazine. Thus, chitosan hydrogelation in the company of tri-ethylene glycol-phenothiazine aldehyde may occur due to the imine constituents, along with the self-assemblage in cluster formations from reticulated nodes [33,34,35].

Furthermore, it is expected that the presence of the tri-ethylene glycol chain in the tri-ethylene glycol-phenothiazine structure will promote the formation of hydrogen bonds with the present chitosan chain, thereby strengthening the hydrogel structure by supplementary mechanical reticulation. Since the tri-ethylene glycol-phenothiazine derivative has moderate water solubility, to ensure the homogeneity of the chitosan/tri-ethylene glycol-phenothiazine system, the imination chemical reaction was accomplished in a solvent blend, using water and acetone. The variation in the molar ratio of the glucosamine parts of chitosan and tri-ethylene glycol-phenothiazine led to hydrogels with diverse degrees of imination [7,12]. The realized hydrogels are apparently soft and transparent materials, which have successfully fulfilled the reversed tube trial, and according to lyophilization, they are solid-state materials with numerous pores spread throughout the entire volume.

### 5.2. Mercury Recovery Ability

As investigated, we know that chitosan and phenothiazine have a proper high affinity for the mercury chemical element, presenting a substantial premise for their amalgamation within a stable system to retain/keep large quantities from a metal of a comparatively raised density [5,10]. Furthermore, the intensive comportment study of xerogel swelling implies the fact that the mercury ions caused the reinforcement/fortifying of the mixed networks accomplished either with chitosan or with phenothiazine, successfully admitting their mercury recuperation fitting. Chitosan and phenothiazine show a high affinity for mercury, presenting good premises; when combining them so that a new network can appear, they are able to retain larger quantities of this specified metallic element [31]. Furthermore, the swelling comportment examination of the xerogels advised us that the mercury ions caused the reinforcement of the chitosan-phenothiazine system, rendering this system suitable for mercury recuperation. It can be easily observed that mercury recuperation depended on the degree of reticulation of the xerogels, along with the concentration of the mixture, idem, etc [10,15]. As assumed, the available specimens were capable of recovering a higher quantity of hydrargyrum from a large concentration of the blend because of the fact that a higher density of Hg ions in the sample was present. When respecting the effect of the level of reticulation, it was noticed that the chitosan-phenothiazine specimens had almost doubled in their retention capability in comparison to clean/pure chitosan.

### 5.3. Equipment and Methods

The spectra in the infrared domain were realized with the help of a spectrometer (FTIR Bruker Vertex 70), operating in the transmission regime, utilizing KBr granules at normal temperature and pressure and a 2 cm^−1^ resolution. Origin8 Pro 8.0 software was utilized to process the recorded spectra. The NMR investigations were executed on the spectrometer (Bruker Avance Neo (400 MHz)) provided with a space/probe-type instrument based on four 5 mm diameter cores and unbiased *z*-axis-gradient detection. Both spectrums, photoluminescence and UV-Vis absorption, were realized by using a spectrophotometer (PerkinElmer LS 55) and an Agilent Cary 60 UV-Vis spectrophotometer, respectively, on the solid specimens. The SEM pictures were produced by using a scanning electron microscope (SEM EDAX—Quanta 200) at a smaller energy of 20 Kev for the electrons [7]. The EDX can be utilized for qualitative and quantitative investigation, permitting us to identify all types of existing elements, like the concentration percentage within the specimen. Moreover, as with conventional SEM procedures, the EDX techniques are nondestructive and necessitate reduced specimen physical preparation, which does not deteriorate the evaluated sample in any way. In this paper, the SEM-EDX spectrum of the 6X-Hg image (Figure 9b) successfully confirmed the presence of mercury in the investigated xerogel specimen.

## Figures and Tables

**Table 1:** Fractal parameter values of an image of 6X-Hg.

FD1	Standard Deviation 1	FD2	Standard Deviation 2	Lacunarity
1.604	±0.2798	1.596	±0.0460	0.0402

## Data Availability

The data used to support the findings of this study cannot be accessed due to commercial confidentiality.

## References

[1] Wu D., Sedgwick A.C., Gunnlaugsson T., Akkaya E.U., Yoon J., James T.D. (2017). Fluorescent Chemosensors: The Past, Present and Future. Chem. Soc. Rev..

[2] Balali-Mood M., Naseri K., Tahergorabi Z., Khazdair M.R., Sadeghi M. (2021). Toxic Mechanisms of Five Heavy Metals: Mercury, Lead, Chromium, Cadmium, and Arsenic. Front. Pharmacol..

[3] Tchounwou P.B., Yedjou C.G., Patlolla A.K., Sutton D.J. (2012). Heavy Metal Toxicity and the Environment. Molecular, Clinical and Environmental Toxicology: Volume 3: Environmental Toxicology.

[4] Bansod B., Kumar T., Thakur R., Rana S., Singh I. (2017). A Review on Various Electrochemical Techniques for Heavy Metal Ions Detection with Different Sensing Platforms. Biosens. Bioelectron..

[5] Kinuthia G.K., Ngure V., Beti D., Lugalia R., Wangila A., Kamau L. (2020). Levels of Heavy Metals in Wastewater and Soil Samples from Open Drainage Channels in Nairobi, Kenya: Community Health Implication. Sci. Rep..

[6] New Limits for Heavy Metals in Food Supplements. https://www.gmp-compliance.org/gmp-news/new-limits-for-heavy-metals-in-food-supplements.

[7] Cibotaru S., Ailincai D., Andreica B.I., Cheng X., Marin L. (2022). TEGylated Phenothiazine-Imine-Chitosan Materials as a Promising Framework for Mercury Recovery. Gels.

[8] Dórea J.G. (2011). Integrating Experimental (In Vitro and In Vivo) Neurotoxicity Studies of Low-Dose Thimerosal Relevant to Vaccines. Neurochem. Res..

[9] Aragay G., Pons J., Merkoçi A. (2011). Recent Trends in Macro-, Micro-, and Nanomaterial-Based Tools and Strategies for Heavy-Metal Detection. Chem. Rev..

[10] Fouda S.R., El-Sayed I.E., Attia N.F., Abdeen M.M., Abdel Aleem A.A.H., Nassar I.F., Mira H.I., Gawad E.A., Kalam A., Al-Ghamdi A.A. (2022). Mechanistic Study of Hg(II) Interaction with Three Different α-Aminophosphonate Adsorbents: Insights from Batch Experiments and Theoretical Calculations. Chemosphere.

[11] Mahmoud M.E., Abdelwahab M.S., Ibrahim G.A.A. (2022). The Design of SnO_2_-Crosslinked-Chitosan Nanocomposite for Microwave-Assisted Adsorption of Aqueous Cadmium and Mercury Ions. Sustain. Chem. Pharm..

[12] Zhang L., Jiao X., Zhang H., He S., Cheng X. (2022). Novel Chitosan–Naphthalimide–Amino Acid Fluorescent Powder for Selective Detection and Removal of Hg^2+^/Hg^+^ and Fe^2+^ in Aqueous Solution. Chem. Pap..

[13] Lin H., Duan Y., Zhao B., Feng Q., Li M., Wei J., Zhu Y., Li M. (2022). Efficient Hg(II) Removal to Ppb Level from Water in Wider PH Based on Poly-Cyanoguanidine/Graphene Oxide: Preparation, Behaviors, and Mechanisms. Colloids Surf. A Physicochem. Eng. Asp..

[14] Saenchoopa A., Klangphukhiew S., Somsub R., Talodthaisong C., Patramanon R., Daduang J., Daduang S., Kulchat S. (2021). A Disposable Electrochemical Biosensor Based on Screen-Printed Carbon Electrodes Modified with Silver Nanowires/HPMC/Chitosan/Urease for the Detection of Mercury (II) in Water. Biosensors.

[15] Michailidou G., Koumentakou I., Liakos E.V., Lazaridou M., Lambropoulou D.A., Bikiaris D.N., Kyzas G.Z. (2021). Adsorption of Uranium, Mercury, and Rare Earth Elements from Aqueous Solutions onto Magnetic Chitosan Adsorbents: A Review. Polymers.

[16] Mandelbrot B.B. (1977). Fractal Geometry of Nature.

[17] Nichita M.V., Paun M.A., Paun V.A., Paun V.-P. (2020). Image Clustering Algorithms to Identify Complicated Cerebral Diseases. Description and Comparison. IEEE Access.

[18] Sarkar N., Chaudhuri B.B. (1992). An efficient approach to estimate Fractal Dimension of textural images. Pattern Recognit..

[19] Karperien A.L., Jelinek H.F., Di Ieva A. (2016). Box-Counting Fractal Analysis: A Primer for the Clinician. The Fractal Geometry of the Brain.

[20] Karperien A., Jelinek H.F., Miloševic N.T. Reviewing Lacunarity Analysis and Classification of Microglia in Neuroscience. Proceedings of the 8th European Conference on Mathematical and Theoretical Biology.

[21] Nichita M.V., Paun M.A., Paun V.A., Paun V.P. (2019). Fractal analysis of brain glial cells. Fractals dimension and lacunarity. Univ. Politeh. Buchar. Sci. Bull. Ser. A Appl. Math. Phys..

[22] Bordescu D., Paun M.A., Paun V.A., Paun V.P. (2018). Fractal analysis of Neuroimagistic. Lacunarity degree, a precious indicator in the detection of Alzheimer’s disease. Univ. Politeh. Buchar. Sci. Bull. Ser. A Appl. Math. Phys..

[23] Popovic N., Radunovic M., Badnjar J., Popovic T. (2018). Fractal dimension and lacunarity analysis of retinal microvascular morphology in hypertension and diabetes. Microvasc. Res..

[24] Postolache P., Borsos Z., Paun V.A., Paun V.P. (2018). New Way in Fractal Analysis of Pulmonary Medical Images. Univ. Politeh. Buchar. Sci. Bull. Ser. A Appl. Math. Phys..

[25] Pereira L.M. (2010). Fractal Pharmacokinetics. Comput. Math. Methods Med..

[26] de Melo R.H.C., Conci A. (2013). How Succolarity could be used as another fractal measure in image analysis. Telecommun. Syst..

[27] Xia Y., Cai J., Perfect E., Wei W., Zhang Q., Meng Q. (2019). Fractal dimension, lacunarity and succolarity analyses on CT images of reservoir rocks for permeability prediction. J. Hydrol..

[28] Paun M.A., Postolache P., Nichita M.V., Paun V.A., Paun V.P. (2023). Fractal Analysis in Pulmonary CT Images of COVID-19-Infected Patients. Fractal Fract..

[29] Paun M.-A., Paun V.-A., Paun V.-P. (2022). A Multifractal Vision of 5-Fluorouracil Release from Chitosan-Based Matrix. Gels.

[30] Paun M.-A., Nichita M.-V., Paun V.-A., Paun V.-P. (2022). Xerogels Morphology Details by Multifractal Analysis and Scanning Electron Microscopy Images Evaluations of 5-Fluorouracil Release from Chitosan-Based Matrix. Gels.

[31] Lungu R., Paun M.-A., Peptanariu D., Ailincai D., Marin L., Nichita M.-V., Paun V.-A., Paun V.-P. (2022). Biocompatible Chitosan-Based Hydrogels for Bioabsorbable Wound Dressings. Gels.

[32] Li Y., Qi X., Chen Y., Wang L., Li Z., Sun J., Jia J. Voxel field fusion for 3d object detection. Proceedings of the IEEE Conference on Computer Vision and Pattern Recognition.

[33] Seidi F., Reza Saeb M., Huang Y., Akbari A., Xiao H. (2021). Thiomers of Chitosan and Cellulose: Effective Biosorbents for Detection, Removal and Recovery of Metal Ions from Aqueous Medium. Chem. Rec..

[34] Czarnobaj K. (2011). Sol–gel-processed silica/polydimethylsiloxane/calcium xerogels as polymeric matrices for Metronidazole delivery system. Polym. Bull..

[35] Wang X., Ahmed N.B., Alvarez G.S., Tuttolomondo M.V., Hélary C., Desimone M.F., Coradin T. (2015). Sol-gel encapsulation of biomolecules and cells for medicinal applications. Top. Med. Chem..

